# Coherence and Entropy of Credit Cycles across the Euro Area Candidate Countries

**DOI:** 10.3390/e23091213

**Published:** 2021-09-14

**Authors:** Adina Criste, Iulia Lupu, Radu Lupu

**Affiliations:** 1 “Victor Slavescu” Centre for Financial and Monetary Research, Romanian Academy, 050711 Bucharest, Romania; a.criste@icfm.ro; 2 Department of International Business and Economics, Bucharest University of Economic Studies, 010404 Bucharest, Romania; radu.lupu@rei.ase.ro; 3 Institute for Economic Forecasting, Romanian Academy, 050711 Bucharest, Romania

**Keywords:** credit-to-GDP gap, coherence, similarity, synchronicity, Central and Eastern European countries, entropy

## Abstract

The pattern of financial cycles in the European Union has direct impacts on financial stability and economic sustainability in view of adoption of the euro. The purpose of the article is to identify the degree of coherence of credit cycles in the countries potentially seeking to adopt the euro with the credit cycle inside the Eurozone. We first estimate the credit cycles in the selected countries and in the euro area (at the aggregate level) and filter the series with the Hodrick–Prescott filter for the period 1999Q1–2020Q4. Based on these values, we compute the indicators that define the credit cycle similarity and synchronicity in the selected countries and a set of entropy measures (block entropy, entropy rate, Bayesian entropy) to show the high degree of heterogeneity, noting that the manifestation of the global financial crisis has changed the credit cycle patterns in some countries. Our novel approach provides analytical tools to cope with euro adoption decisions, showing how the coherence of credit cycles can be increased among European countries and how the national macroprudential policies can be better coordinated, especially in light of changes caused by the pandemic crisis.

## 1. Introduction

Lending activity, which is subject to medium-term fluctuations, is one of the determining factors influencing financial stability, with excessive growth in lending activity over a period being an important signal of risk accumulation. Under the European economic integration process, financial stability and the dynamics of financial activity have increased importance. In addition to the traditional criteria of nominal economic convergence, euro area candidate countries should also take into account how their economies are prepared to join the monetary union. The synchronization of business cycles is already a much debated issue as a criterion for achieving an optimal monetary area, both theoretically and in practice, as discussed in many empirical studies; however, the synchronization of financial cycles in a monetary area is a relative recent topic. The global financial crisis has been a key factor in the increasing interest in the dynamics of lending and the financial cycle, including in European countries.

The credit cycle, which is a common way to empirically measure the financial cycle [1], is an important element that can explain the differences between countries both in terms of economic growth and stability, as well as the effects of political decisions. Moreover, Borio [1] emphasizes the importance of understanding the manifestation of the financial cycle, which is best evidenced by fluctuations in lending activity and property prices, as a premise for understanding fluctuations in economic activity and political challenges. The financial cycle was closely linked to increases in financing and intermediation in the advanced economies in the 1970s, which caused severe recessions, boom and bust cycles, and financial instability, and also to an exponential increase in cross-border lending [2].

At the level of a monetary area, the analysis of the coherence of economic and financial cycles is particularly relevant, given the conditions of the single monetary policy regime. Differences between countries in terms of business cycles increase the need for the central bank to act in accordance with the “one-size-fits-all” principle for monetary policy decisions, given that the same decision produces different effects. In this way, the divergence between economic and financial cycles can be deepened, affecting the stability of the monetary area. Moreover, the financial cycle can affect the process of convergence in a monetary area by misallocating resources to less developed countries in the union if boom–bust cycles occur, as reflected by very large amplitudes. In this regard, Oman [2] points out that the financial cycle could have played an important role in the real economic divergence between the euro area member countries since the introduction of the euro. As a result of the boom–bust lending cycles in the peripheral countries of the Eurozone, resources have been misallocated in these economies, with productivity gains being affected.

In addition to the differences between business and financial cycles, there is also the problem of differences among countries and within them, because they can create tensions and can affect the objectives of macroeconomic stabilization and financial stability.

The synchronization of financial cycles is important in the process of joining a monetary area, in implementing the macroprudential policy, and in the relationship between the macroprudential authorities at the national level and those at the level of the monetary area.

A relatively recent study by Samarina et al. [3] addressed the issue of the coherence of financial cycles within the euro area, although it would be interesting to address this issue for euro area candidate countries, given that they are in the process of joining the monetary union.

Based on the above observations and the importance of the coherence of financial cycles within a monetary area, this article aims to determine the degree of coherence of credit cycles in euro area candidate countries with the euro area credit cycle, which is taken as a reference. As described in the literature [3], the terms financial cycle and credit cycle are frequently used interchangeably, and for this reason we also adopt this approach, without excluding other empirical estimation methods; however, we often use the credit cycle wording to emphasize the chosen estimation method.

After estimating the credit cycles in the selected countries and in the euro area (at aggregate level) by using the Hodrick–Prescott filter, similarity and synchronicity indicators are calculated in the selected countries for the period 1999Q1–2020Q4. The investigation of the dynamics of these variables is further developed by using a set of entropy measures designed for certain time series. Our objectives are three-fold: first, we compute entropy measures for the filtered individual series of credit-to-GDP gap variables for the six euro area candidate countries addressed in our analysis (the block entropy and the entropy rate); second, we compute measures of entropy (transfer entropy) for the same variables in combination with the filtered credit-to-GDP indicator for the euro zone level; third, we estimate a Bayesian entropy measure for the similarity indicators of credit cycle gaps with respect to the euro zone. 

The results show that a long period of low entropy before the commencement of the financial crisis matches the period in which the coherence of credit cycles of the euro area candidate countries is lower. After the crisis, the entropy is heightened, along with the degree of credit cycle coherence, particularly in terms of synchronization. We provide evidence of a high degree of heterogeneity in the dynamics of these variables and develop an analysis toolkit that is necessary to support euro adoption decisions. This data-driven set of indicators will enhance the current convergence gauges and provide new perspectives on the success of euro adoption scenarios.

In this article, we present the main landmarks from the literature in Section 2, while descriptions of the methodology and data used are given in Section 3. The obtained results are presented and interpreted in Section 4, while the main conclusions of the study are presented in Section 5.

## 2. Literature Review

The first studies on financial cycles were conducted by Borio et al. [4] and Borio et al. [5], although concerns have intensified since the onset of the global financial crisis. Come studies have addressed the issue of how to measure financial cycles, while others have analyzed the degree of synchronization in a group of countries [6,7], including at the level of a monetary area [3,8]. In addition, some papers have analyzed the synchronization between business and financial cycles. Among these, we mention the work of Oman [2], who focused on developed European countries, namely those that are in the euro area, as well as the work of Miteski and Georgievska [9], who analyzed emerging European countries.

A number of recent studies involving the measurement of the financial or credit cycle have differed in terms of the indicators used (credit-to-GDP or real credit in log) and the method used to obtain the credit cycle; thus, similar methods are used to measure the credit cycle to those used to measure business cycles, either by applying the turning points method [10] or frequency-based (band pass) filters [11]. Other studies have also used more complex methodologies, as mentioned by Schüler et al. [6] and Borio et al. [12].

Among the studies dedicated to the analysis of financial cycles, it is important to mention those that have analyzed European countries as benchmarks for this article. The paper by Samarina et al. [3] was particularly noteworthy, which aimed to find out whether the adoption of the euro has led to convergence of financial cycles between member states. The analysis took into account 16 Eurozone member states, including new member states (Slovakia, Slovenia, Malta, and Estonia), for a period of 25 years (1990–2015). The lending cycle was broken down into three components: total bank credit, household mortgage lending, and non-financial business loans. The authors concluded that in recent decades, both mortgage and corporate loan cycles have diverged. The study emphasized the importance of differentiating by types of credit to gain knowledge of the credit cycle, along with the importance of differentiating the transmission channels of the effects of euro adoption.

For the European Union countries, Stremmel and Zsámboki [7] noted that the amplitude of the financial cycle is largely determined by the structural characteristics of the financial sector in these states, namely the degree of concentration, share of foreign banks in the banking system, level of lending, the structure of bank loans, as well as financial integration.

Analyzing the evolution of financial and business cycles in the period 1971–2015 (on the basis of quarterly data) for the founding member countries of the Eurozone (except Luxembourg), Oman [2] showed that indigenous financial cycles (specific to each country) tend to be much broader than business cycles, noting the concordance with the results obtained by Drehmann et al. [10] and Galati et al. [13]. Additionally, during the period under review, the synchronization of financial cycles in euro area countries was weaker than that of business cycles. After the introduction of the euro, the degree of synchronization of the business cycles as an average measure of the euro area countries increased over time, while the synchronization of the financial cycles decreased on average.

Regarding the characteristics of financial cycles in euro area countries, recent results [2,6] have shown the existence of a high level of heterogeneity of financial cycles between European countries. On the other hand, Oman [2] pointed out that during the financial crisis, the synchronization of the financial cycle increased on average for their sample, reinforcing the observation made by Stremmel and Zsámboki [7], namely that the euro area countries have a lower degree of divergence, while in normal periods that financial cycles have a lower degree of synchronization.

The heterogeneous nature of financial cycles is also noticeable for other monetary areas. For example, by analyzing the issue of the coherence of financial cycles within the West African Economic and Monetary Union (WAEMU), Gammadigbe [8] showed that during the period 2005Q1–2020Q4, the national financial cycles were heterogeneous in terms of both duration and amplitude, with no convergence of financial cycles.

Some of the important benchmarks in the literature for our research are the methods used to measure the coherence of financial cycles. Aikman et al. [11] used the standard correlation coefficient of credit deviation, while Meller and Metiu [14] applied a concordance index. As mentioned by Samarina et al. [3], the disadvantage of these methods is the fact that the two dimensions of a cycle (amplitude and frequency) are not considered separately.

Regarding the issue of business cycle coherence, the studies by Harding and Pagan [15] and Mink et al. (2011) are good benchmarks for measuring the coherence of financial cycles, given that the two dimensions that define the coherence or concordance of cycles—synchronization and similarity—are assessed separately using the proposed methodologies. While cycle synchronization refers to the frequency of fluctuations, similarity is defined by the amplitude of the fluctuations. The difference between the two methodologies lies in the chosen indicator—while Harding and Pagan (2006) use the level of GDP to determine the business cycle, Mink et al. [16] use the deviation of the GDP (output gap). 

The importance of financial cycles can also be seen from the perspective of the application of macroprudential instruments meant to correct possible threats to financial stability, as signaled by the evolution of the financial cycle [1]. In this sense, Samarina et al. [3] emphasized the need to capitalize on macroprudential policy instruments, given that the results of their study showed a fairly large divergence of lending cycles in the euro area. Oman [2] showed that the macroprudential instruments used for correcting the financial cycle complement those intended to correct the business cycle, especially since the two are not always synchronized.

The role played by entropy in financial research has been shown by Zhou, Cai, and Tong [17]. The authors emphasized the use of information and probability entropy as important instruments for portfolio selection issues and asset pricing, although they encouraged a wider application of different types of entropy in finance, as the results were mostly consistent with the original models, which opened up new investigation directions. With the aim of constructing a network of influences in the global financial sector, Sandoval [18] performed transfer entropy measures, showing that the 197 largest financial companies are related and obtaining stricter results than those that were obtained when the companies were analyzed through correlations. In order to compute a system credit factor, Xu and Ren [19] used the cross entropy, with their results supporting the idea of applying entropy measures to study credit cycles. In another study, the synchronization of business cycles was analyzed via the implementation of a pairwise maximum entropy model for the G7 member countries [20], showing that this is an appropriate method for small economic systems.

From the analysis of the literature, it is noted that most of the previous studies have taken into account countries with advanced economies. From this point of view, this article expands the research area in this field. Moreover, the topic of cycle coherence is relevant not only in the case of a monetary union such as the euro area, but also for countries that are about to join a monetary union, namely candidate countries for the euro area.

## 3. Materials and Methods

The analysis of the financial cycle in the euro area candidate countries took into account bank lending, considering the predominance of this sector in these countries, namely Bulgaria, Croatia, Czech Republic, Hungary, Poland, and Romania. 

Quarterly data were extracted from the central banks’ statistics for the selected countries (regarding the bank credit to the private non-financial sector) and from the Eurostat database (for GDP) for the period 1999Q1–2020Q2. Data for the euro area at the aggregate level were taken from the BIS database.

This paper had three research topics:

(1) Assessing the trajectory of credit cycles in euro area candidate countries;
(2) Assessing the coherence levels of the credit cycles in these countries with the euro area credit cycle;
(3) Performing an entropy analysis.

### 3.1. Estimation of the Credit Cycle

Statistical data were taken from the Eurostat and central bank databases for the period 1999–2020. In our analysis, bank credit was considered, given that in the selected countries the banking system is dominant in relation to the non-banking system [9]. Quarterly and seasonally unadjusted GDP data were taken from the Eurostat database, while the volume of bank lending to the private non-financial sector was calculated based on data provided by the central banks of the euro area candidate countries. In order to avoid the transition period, marked by major changes in the profiles of the selected economies, the time interval of 1999Q1–2020Q4 was chosen.

The credit cycle was estimated using the Hodrick–Prescott (HP) filter method. The HP filter estimates the credit cycle for euro area candidate countries by computing the credit-to-GDP gap. 

The HP filter isolates the cyclical component (*c_t_*) from the trend (*Trend_t_*) of a non-stationary series

(1) $${\left(y_{t}\right)}_{t=0}^{n}$$

so that

(2) $$y_{t}=Trend_{t}+c_{t}.$$

This isolation method is based on a minimization problem of the form:

(3) $$\underset{T_{1},\ldots ,T_{1}}{min}\left\{\sum_{t=1}^{n}{\left(y_{t}-Trend_{t}\right)}^{2}+\lambda \sum_{t=2}^{T-1}[(Trend_{t+1}-Trend_{t}c_{t})-(Trend_{t}-Trend_{t-1}){]}^{2}\right\}$$

The adjustment parameter or smoothing factor *λ* corrects the deviations from the trend and is a factor chosen by the user, depending on the frequency of the data. The following values are used for the business cycle: *λ* =100 (for annual data), *λ* = 1.600 (for quarterly data), *λ* = 14.400 (for monthly data).

According to the literature, the indicator that reflects the credit cycle very well is the credit-to-GDP gap or the deviation of the Basel indicator from the long-run statistical trend, which is performed following three steps:

(1) Computing the credit-to-GDP percentage ratio in year *T*;
(2) Estimating the credit-to-GDP trend;
(3) Finding the “Basel gap”.

Equation (4) describes the credit-to-GDP ratio at the quarterly level, *q_t_*, as the ratio between the bank lending to the private non-financial sector (firms and households) in the *q_t_* quarter and the cumulated GDP for four quarters prior to the quarterly credit calculation date, according to the methodology used by the Bank for International Settlements (BIS):

(4) $${\left(Credit/GDP\right)}_{q_{t}}=\frac{C_{bp}\left[q_{t}\left(T\right)\right]}{\sum_{n=t}^{t+3}GDP_{n}\left(T-1\right)}*100,$$

where:

- *q_t_* = quarter *t*, *t* ∈ {1; 2; 3; 4};
- *T* = year *T*, *T* ∈ {1999; 2000; … 2020};
- *C_bp_* = bank credit to the private non-financial sector
- *GDP_n_* = quarterly and seasonally unadjusted GDP data.

For this estimation, a higher adjustment parameter was used for the credit cycle (*λ* = 25,000) compared to the business cycle case, in which the parameter corresponding to the quarterly period was lower (*λ* = 1600). This was justified by the fact that the financial cycle (with the credit cycle being a major component of the financial cycle) generally lasts about four times longer than the business cycle. On the other hand, BIS recommends a much higher parameter (*λ* = 400.000) for longer time series over 20 years. Moreover, recent research [21] confirmed the need to use a lower smoothing factor (*λ*) than that proposed by the BIS for economies with shorter financial cycles or lower levels of financial development (short financial depth), such as the emerging European countries or the transition countries. For the euro area, taken as a reference here, the smoothing factor is the one recommended by the BIS.

The credit-to-GDP gap is measured in GDP percentage units, according to the formula:

(5) CreditGDPgapqt=−1−RCreditGDPqtTrendCreditGDPqt,

where:

(6) RCreditGDPqt

is the actual credit-to-GDP ratio in *q_t_* and:

(7) TrendCreditGDPqt

is the long-term trend for credit-to-GDP ratio in *q_t_* (estimated according to the HP filter for *λ* = 25.000).

### 3.2. The Credit Cycle Coherence

The credit cycle coherence is estimated following the methodology used by Mink et al. [16] considering the two components, i.e., the degrees of synchronization and similarity, while the benchmark used for this measurement is the euro area, as the region in which the countries from our sample are to be integrated.

We note *c_i_(t)* as the credit deviation for country *i* in period *t* (quarter) and *c_r_(t)* as the credit deviation for the euro area, taken as reference *r*, in the same period *t*.

The degree of synchronization between the credit cycle of a country *i*, *c_i_*(*t*), and the reference (*c_r_*(*t*)) at time *t* is given by the formula:

(8) $$syn_{ir}\left(t\right)=\frac{c_{i}\left(t\right)c_{r}\left(t\right)}{\left|c_{i}\left(t\right)c_{r}\left(t\right)\right|}.$$

The value of the coefficient, ±1, indicates the direction of the credit cycle for *i* with respect to the reference. The positive value shows synchronization, while the negative value shows desynchronization (the two trajectories move in opposite directions). The degree of synchronization over 1999Q1–2020Q4 is calculated as the arithmetic mean of the values registered during this period.

The degree of similarity (*sym_ir_*(*t*)) is the difference in amplitude between the credit deviation for *i* and the reference deviation, according to the formula:

(9) $$sym_{ir}\left(t\right)=1-\frac{c_{i}\left(t\right)-c_{r}\left(t\right)}{\frac{1}{n}\sum_{i=1}^{n}\left|c_{i}\left(t\right)\right|},\;sym_{ir}\left(t\right)\in [1-n;\;1]$$

A value of 1 shows that the two compared credit cycles (of country *i* and of reference *r*) have the same amplitude. The value of 1 − *n* is recorded when the values of the two indicators (country *i* and reference *r*) have opposite signs and the gap for all other countries is zero.

### 3.3. The Entropy Approach

As recently emphasized [22], the application of the entropy approach in economics is considered a ”factor of progress”, while mixing standard economic methodological approaches with natural science investigation tools is perceived as a factor for economic research development [23]. 

Block entropy is based on Shannon entropy [24], which is implemented on time series with *k* histories and is calculated as follows:

(10) $$H\left(X^{\left(k\right)}\right)=-\underset{x_{i}^{\left(k\right)}}{\sum }p\left(x_{i}^{\left(k\right)}\right)log_{2}p\left(x_{i}^{\left(k\right)}\right),$$

where *X* is a random variable, *x_i_* is the iteration *i* of the time series described by the variable *X*, and *k* denotes the histories of the time series (the block size). The probability of observing $x_{i}^{\left(k\right)}$ is itemized $p\left(x_{i}^{\left(k\right)}\right)$. According to this specification, Hlavackova-Shindler et al. [25] interpreted the Shannon entropy indicator as the “quantity of surprise one should feel upon reading the result of a measurement”, which is directly proportional to uncertainty. For the application on the time series, we will interpret this indicator as the level of surprise to expect at each moment in time.

The entropy rate, also known as the source information rate, represents the entropy of the time series, in this case conditioned by the *k*-histories. In other words, it measures the quantity of needed information in order to display the $X^{\left(k\right)}$ observations. The global entropy rate is obtained from the average of the local entropy:

(11) $$H_{X}\left(k\right)=h_{X,i}{\left(k\right)}_{i}=\sum_{x_{i}^{\left(k\right)},\;x_{i+1}}p\left(x_{i}^{\left(k\right)},\;x_{i+1}\right)log_{2}\frac{p\left(x_{i}^{\left(k\right)},\;x_{i+1}\right)}{p\left(x_{i}^{\left(k\right)}\right)},$$

as suggested by Cover and Thomas [26].

Two decades ago, Schreiber [27] led in the transfer entropy to measure the information that is transferred between the source and destination, taking into account the background of the system, denoted with *W* [28]. A local time variant is used to define the transfer entropy:

(12) $$t_{X\to Y,W,i}\left(k\right)=log_{2}\frac{p(y_{i+1},\;x_{i}|y_{i}^{\left(k\right)},\;W_{\left\{1,i\right\}}),\ldots ,W_{\left\{l,i\right\}}}{p\left(y_{i+1}|y_{i}^{\left(k\right)},\;W_{\left\{l,i\right\}}\right)p(x_{i}|y_{i}^{\left(k\right)},\;W_{\left\{1,i\right\}},\ldots ,W_{\left\{l.i\right\}})}.$$

The methodology for Bayesian entropy was previously described by Lupu et al. [29], following the study by Archer, Park, and Pillow [30]. In the estimation process of the Bayesian entropy (denoted *H*), we take into account that this is a deterministic function of a discrete distribution (π), which is influenced by parameter θ. Given that *p*(π) is a prior distribution, *p*(π|x) represents the posterior distribution over π, *p*(x|π) designates the discrete likelihood, and *H* has a deterministic relation with π, we may consider the following expressions:

(13) $$p\left(H|\pi \right)=\delta \left(H+\sum_{i}\pi_{i}log\pi_{i}\right)$$

(14) $$\hat{H}\left(x\right)=E\left[H|x\right]=\int H\left(\pi \right)p(H|\pi )p(\pi |x)d\pi ,$$

where the last expression is the form for Bayes’ least squares estimators.

The computation of these entropy indicators required the discretization of our data, for which we followed the method of Archer, Park, and Pillow [30]. The dynamic values were obtained by using a rolling window of four observations, which was equivalent to one year, given that we used quartrly data.

## 4. Results 

**Figure 1:**
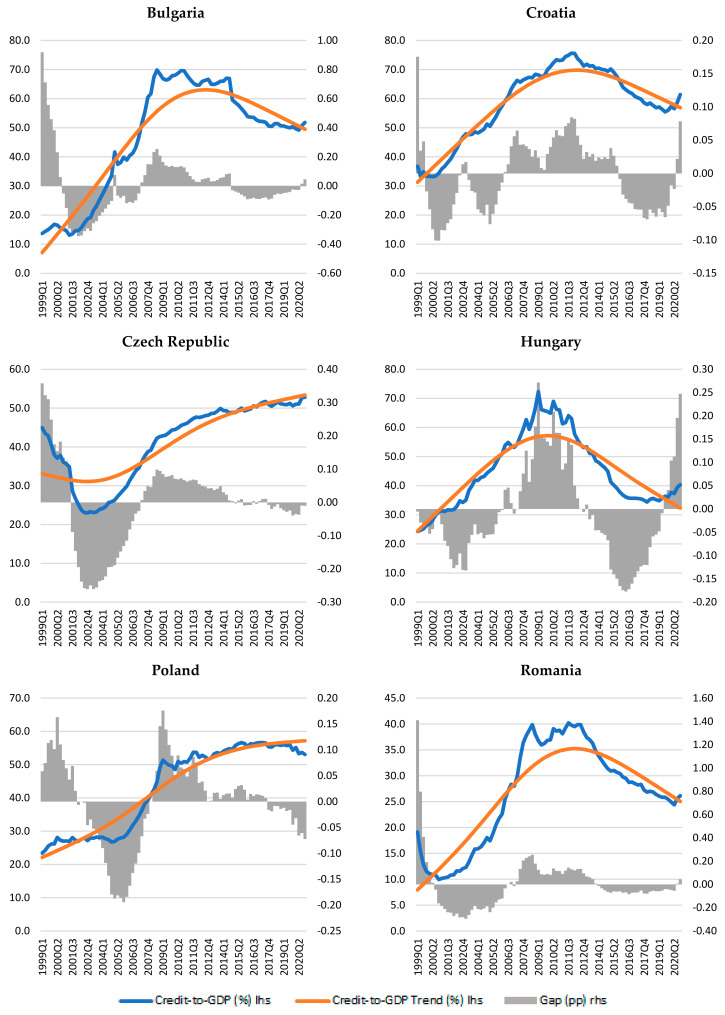
The bank credit cycle in the euro area candidate countries.

The results obtained based on the HP filter method showed a rather varied picture of credit cycles in the countries considered (see Figure 1).

According to these charts (Figure 1), similar curves can be seen for Bulgaria, Croatia, Hungary, and Romania in an ascending–descending pattern, while on the other hand the Czech Republic and Poland show an ascending pattern throughout the period, especially after 2001.

Croatia, Poland, and the Czech Republic show small deviations from the trend throughout the period under review. In Hungary, there are higher levels of credit-to-GDP gaps after 2007 as compared to those recorded in the first part of the analyzed interval. 

Although overall the euro area candidate countries do not have synchronized credit trajectories, it can be observed that the periods in which the credit-to-GDP gaps are positive re relatively the same for Bulgaria, Romania, and the Czech Republic (1999–2001 and 2007–2014, respectively), provided that the amplitudes are different. The highest amplitudes are shown for Bulgaria and Romania, especially in the first year (see Figure 1).

**Figure 2:**
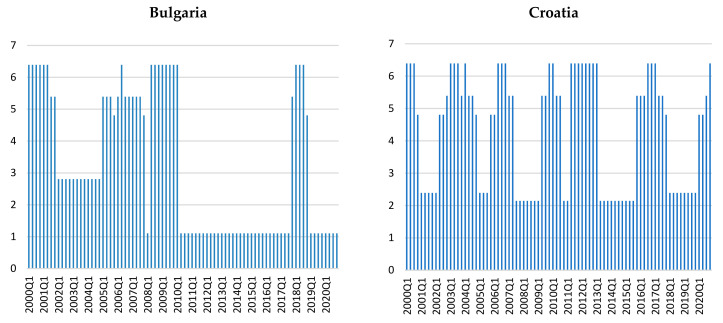

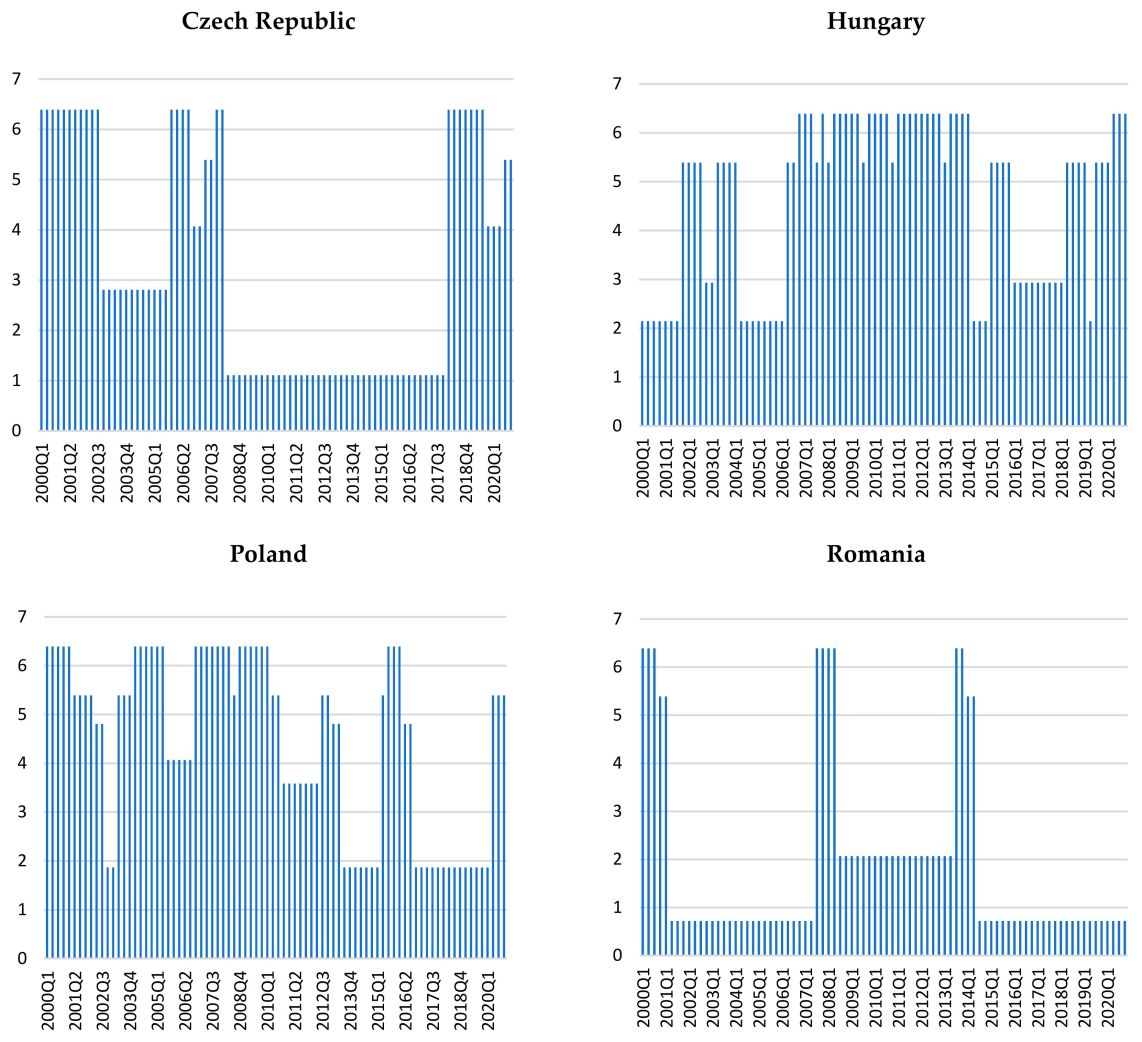
Shannon entropies for filtered credit gaps.

The Shannon entropies computed over the preceding year (previous four observations) for each of the six countries with filtered credit gaps provide information about the level of surprise to expect at each moment in time (Figure 2). We note that there are approximately three main regimes for Croatia and Romania, four main regimes for Bulgaria and Czech Republic, and even more for Hungary and Poland. 

The most volatile series belongs to the Croatian credit-to-GDP gaps, while in Romania the stable stages are longer. We notice that these regimes do not exhibit a simultaneous structure, i.e., the credit cycles tend not to be very entropic or less entropic in the same time across these countries. For instance, the COVID-19 period tends to produce large levels of entropy in Croatia, the Czech Republic, Poland, and Hungary, but not so much in Bulgaria and Romania, which seem to continue the dynamics described by the HP filter. If we connect the concept of entropy with the idea of uncertainty, then we can say that, except for Bulgaria and Romania, the countries in our sample exhibit higher uncertainty during the pandemic period.

Further data can be revealed by observing the entropy rate, which shows the amount of information needed to describe the values of a certain variable given a sequence of observations (rolling window) from its past. The size of the rolling window is four in our case, which extends to the length of one year. Under this specification, the high levels of this indicator will correspond to situations where uncertainty existing in the data series is elevated, depicting a moment when the time series are impacted by factors that are suddenly activated or deactivated. 

**Figure 3:**
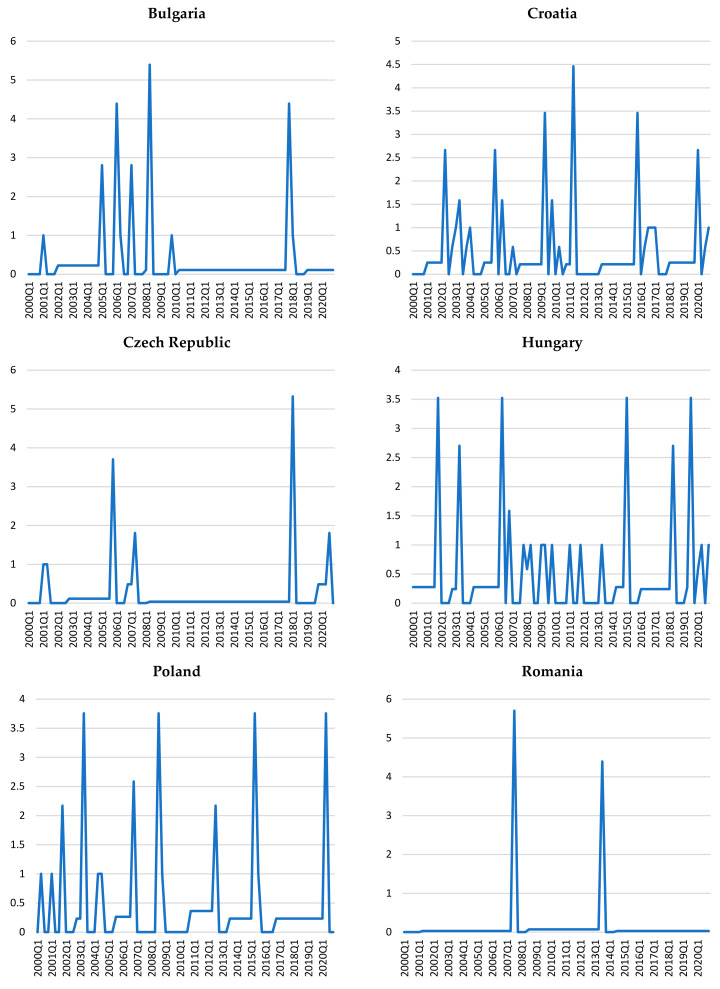
The entropy rates for filtered credit gaps.

We note that for each of the series of gaps from credit-to-GDP variables, the values are quite volatile (Figure 3). 

High levels of volatility are especially observed for Croatia, Hungary, and Poland. Romania, on the other hand, exhibits only two spikes in the dynamics of its corresponding series, revealing a rather steady entropy rate that could be caused by the fact that it has the lowest credit-to-GDP ratio among the countries in our sample. 

As in the previous analysis (the Shannon entropy), no simultaneity effects can be observed for the large values of the entropy rate; however, except for Romania and Bulgaria, the pandemic period exhibits either spikes or increased volatility of this indicator.

Noting the scale of the vertical axes for these charts, we can conclude that volatility compensate for jumps. In other words, the large volatility (present for Croatia, Hungary, and Poland) also reduces the large values, as these variables do not show such large extremes as the other three countries (Figure 3).

The coherence of the credit cycles in terms of synchronicity and similarity is displayed both in Table 1, as the average values over 1999Q1–2020Q4 for these two variables, and in Table 2, with the values computed for two sub periods, i.e., before and after the global financial crisis.

According to the data displayed in Table 1, the highest degrees of synchronicity and similarity with the euro area credit cycle over 1999–2020 are noted for Croatia, while the lowest levels of similarity (negative levels) are recorded for Bulgaria, Romania, and Hungary.

Overall, the countries have relatively good synchronization with the euro area compared to the level of similarity, which is quite low. The large differences in amplitudes between countries in terms of lending activity can be explained by the lower levels of economic and financial development of these countries compared to the euro area.

An analysis at the level of the two-time intervals (before and after the onset of the global financial crisis) could point out the extent to which these countries are going through a process of convergence in lending activity.

As can be seen in Table 2, before the onset of the global financial crisis, Hungary shows the highest level of credit cycle coherence with the euro area, both in terms of synchronicity and similarity. It is noteworthy that after 2008, Hungary suffers a significant desynchronization from the reference and a reduction in the level of similarity.

Although in the second part of the sampled period, Croatia does not register significant increases of the two indicators, it maintains its best position among the six states. Instead, Poland, the Czech Republic, and Bulgaria show improvements in both indicators during this period. Romania shows better results in terms of synchronicity and poorer results in terms of similarity with the euro area.

The better synchronization of credit cycles in the aftermath of the global financial crisis is consistent with the observation made by Omen [2] and Stremmel and Zsámboki [7] for euro area countries, namely that the financial cycle synchronization increases in times of financial stress.

**Figure 4:**
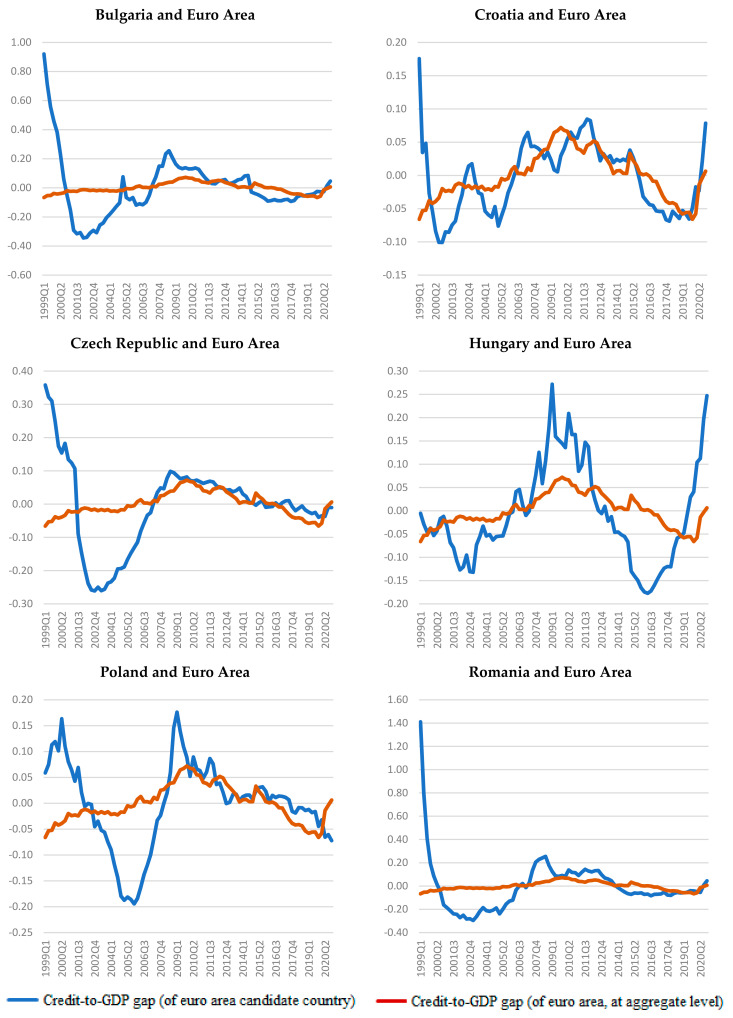
Credit cycles for 1999Q1–2020Q4.

These observations are also highlighted in Figure 4, where we compare the credit cycles for each of the six countries with the euro area credit cycle. It is noted that the credit cycles in all six countries have a longer time of synchronization with the Eurozone after the global financial crisis outbreak (2007–2014). In the Czech Republic and Poland, the synchronization periods are longer at nine years (2006–2015) and ten years (2008–2017), respectively. 

Moreover, after the global financial crisis, the narrowing of the credit cycle gaps in our sample compared to the reference (euro area) is emphasized, especially for the Czech Republic and Poland and less so for Bulgaria and Romania, given the wider variation range of the credit-to-GDP gaps for these two countries (see Figure 4).

An insight into the extent to which the dynamics of the gaps in the credit-to-GDP rates for each individual series depends on the corresponding euro area values is reflected by the transfer entropy measure. As previously stated, this indicator measures the information that is transferred between the source and destination, taking into account the background of the system. Here, we consider the source to be the series of credit-to-GDP gaps for the Eurozone and the destination to be the corresponding variables for each of the countries. 

In keeping with this paradigm, we interpret the transfer entropy measure as the level by which the uncertainty reduced for the credit-to-GDP gaps for each of the six countries through knowledge of the past values of the credit-to-GDP gap for the Eurozone. From this perspective, we can conjecture that the positive values reflect reductions in uncertainty, while the negative values expose increases in uncertainty.

**Figure 5:**
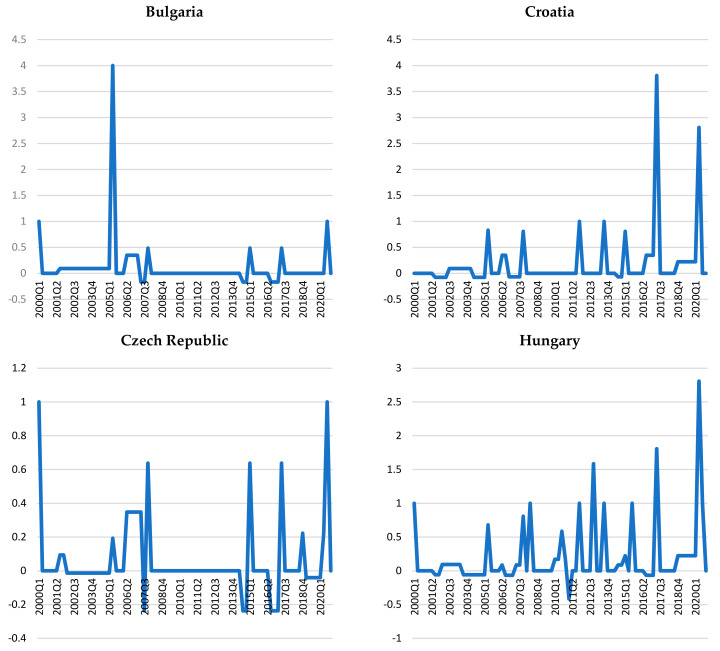

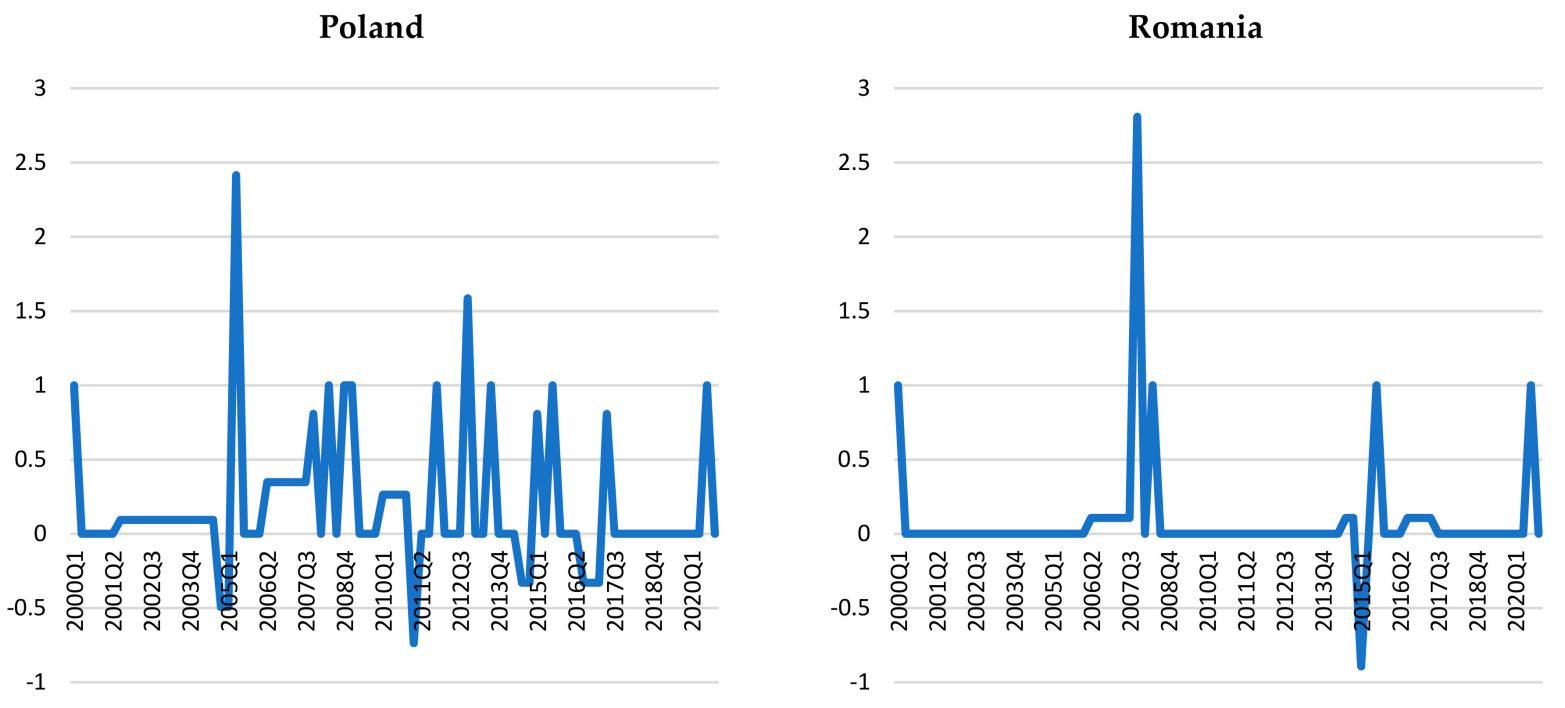
The transfer entropy values for filtered credit gaps in correspondence with euro area values.

The series with very few and lower negative values are those reflecting the credit-to-GDP gaps in Croatia, which is a country whose credit cycles seems to feature a high level of resemblance with the Eurozone (Figure 5). The negative values are also a sign of idiosyncrasy. The fact that the Eurozone induces uncertainty in the evolution of the credit-to-GDP gaps reflects the particularities of each of these countries, their cultural interpretation of credit, and their propensity to use credit for business development. We note that the negative values are not simultaneous across countries, which is another sign of these idiosyncratic effects.

The spike at the end of the series corresponds to the pandemic period. These large values reveal that the credit-to-GDP pattern in the Eurozone is informative for the evolution of the credit cycles in all countries in our sample (Figure 5). 

**Figure 6:**
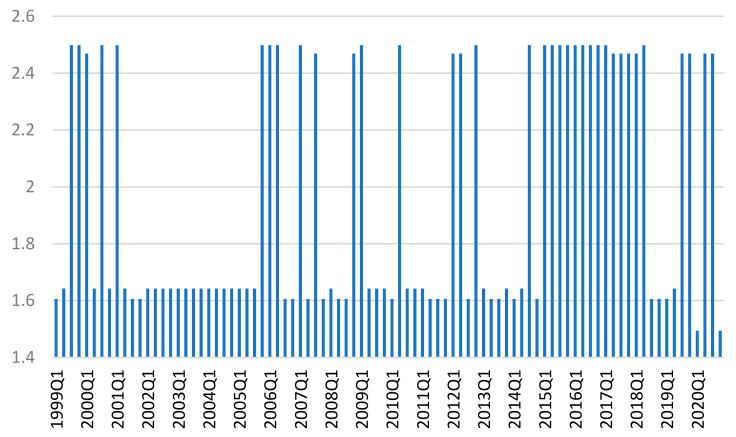
The Bayesian entropy values across all the six similarity measures.

Another perspective of the extent to which these six countries have similar credit cycles with the Eurozone is revealed by the measurement of the Bayesian entropy across all six similarity measures at each point in time. We note the existence of two regimes in the evolution of this system, with a long period of low entropy just before the large financial crisis and a long period of large entropy during 2015–2018 (Figure 6). The volatility in the last two years can first be attributed to the rather calm period in 2019 and to the start of the pandemic episode in 2020.

It should be noted that the long period of low entropy before the onset of the financial crisis corresponds to the period in which the coherence of credit cycles of the euro area candidate countries is lower, while after the crisis the entropy is increased, as well as the degree of the credit cycle coherence, especially in terms of synchronization. 

The obtained results for our sample can also be seen through the lens of the differences regarding the monetary regime and the monetary policy strategy and the institutional arrangements regarding the macroprudential policy and their reporting and responsibility towards this policy. The main objective of the central banks from these countries is price stability, although the path is adapted differently to the domestic economic situation and to the history of macroeconomic imbalances. If this objective is pursued on the basis of an inflation targeting strategy for the monetary authorities of the Czech Republic, Hungary, Poland, and Romania, the emphasis is on the stability of the national currency for the central banks of Croatia and Bulgaria. With the exception of Bulgaria, which has a fixed exchange rate regime against the euro, the other countries have a more or less flexible exchange rate regime. Regarding the macroprudential policy, the profiles of the central banks are even more diverse. On the other hand, these countries are exposed to a number of common challenges they face from their current status as European Union member countries or as candidate countries for the euro area.

## 5. Discussion and Conclusions

As a general conclusion that emerges from this study, the heterogeneous character of lending activity across the euro area candidate countries is highlighted, both in terms of the entropy of credit cycles and their coherence in relation to the euro area reference.

Based on the entropy measurement results, Croatia has the most volatile series (Shannon entropy) with highest level of volatility, although lower entropy rate values, as can be seen from the credit-to-GDP gap results obtained based on the HP filter method. In Poland and Hungary, the Shannon entropy also shows high levels of volatility. Unlike Poland, which has higher levels in the first half of the sampled period, Hungary shows higher entropy, especially after 2006.

Over the entire period, better coherence in terms of both synchronicity and similarity with the euro area credit cycle is shown for Croatia, while the lowest levels of similarity (negative levels) are shown for Bulgaria, Romania, and Hungary; however, separate analysis over the two-time intervals (before and after the onset of the global financial crisis) shows certain differences in the dynamics of this coherence of the countries towards the euro area. In this regard, before the onset of the global financial crisis, Hungary shows the highest level of credit cycle coherence with the euro area, both in terms of synchronicity and similarity, although after 2008 it suffers significant desynchronization from the reference area and a decrease in the level of similarity, which is more accentuated after 2014 and during the pandemic. In Hungary, the role of the central bank was neutral in adopting macroprudential policies in the lead up to the global financial crisis, while the authorities responsible for banking supervision considered “expansive” lending to be a normal reaction to the convergence process with the European Union. Although there were concerns about the expansion of foreign currency lending and mortgages, the authorities did not impose restrictions for political and social reasons. Macroprudential measures were applied more intensely during the crisis period than before the crisis in response to growing concerns about the risks to financial stability associated with foreign currency mortgages, which blocked the banks’ operations.

Although in the second part of the sampled period, Croatia, a country with a high degree of euroization in the economy, does not show significant increases of the two indicators, it still holds the best position among the six states.

The data on the entropy transfer show that the credit cycle in Croatia has a high level of resemblance with the euro area, confirming its better coherence, as reflected by having the highest levels of similarity and synchronicity. It is noted that the credit cycle in Croatia has a stable path concerning the coherence with the euro area throughout the period.

It is noted that the credit cycles in all six countries have longer periods of synchronization with the Eurozone during the global financial crisis and in the first years after its onset (2007–2014). The better synchronization of the credit cycles, as perceived in the aftermath of the global financial crisis, confirms the idea mentioned in the literature, namely that financial cycle synchronization increases in times of financial stress. Overall, the countries show relatively better synchronization with the euro area than similarity. Simultaneously, during the 2007–2013 period, as compared with the period before the financial crisis, the use of macroprudential instruments is more intense, reflecting an increase in central bank activism in this regard, counteracting the effects of the crisis. This intensification is more evident for the countries that had already had similar experiences (Croatia, Bulgaria, Poland, and Romania). In addition, greater involvement is shown for Hungary as a result of the increasing role of the central bank in its macroprudential policy; however, the Czech National Bank maintained its relatively “neutral” position on the use of macroprudential measures.

This topic will remain of interest in the next future, considering not only the process of preparation for the accession to the euro area for these six countries, but also the manifestation of the COVID-19 crisis, which is supposed to change the trajectory of the financial cycles.

Credit cycle coherence with the euro area is not automatically attained by simply joining the monetary union. This observation has been mentioned in recent studies, according to which the adoption of the euro has not led to the synchronization of financial cycles in the euro area. Assuming that macroprudential policy is one of the main macroeconomic policy instruments that can influence the dynamics of the financial cycle, it is expected that better coherence of credit cycles in a monetary area will be obtained through better coordination of national macroprudential policies.

The obtained results should also be analyzed in comparison with the other components of the financial market, namely the financial assets market and the real estate market.

## Figures and Tables

**Table 1:** The credit cycle coherence levels for euro area candidate countries with the euro area for 1999Q1–2020Q4 ^1^.

	Synchronicity	Similarity
Croatia	0.773	0.638
Romania	0.545	−0.072
Bulgaria	0.523	−0.114
Hungary	0.500	−0.043
Czech Republic	0.455	0.274
Poland	0.364	0.321

^1^ The means of values registered over 1999Q1–2020Q4.

**Table 2:** The credit cycle coherence levels of euro area candidate countries with the euro area, before and after the onset of the global financial crisis ^1^.

1999–2007			2008–2020		
	Synchronicity	Similarity		Synchronicity	Similarity
Hungary	0.833	0.692	Croatia	0.875	0.650
Croatia	0.667	0.620	Bulgaria	0.708	0.162
Romania	0.500	−0.355	Poland	0.708	0.547
Bulgaria	0.278	−0.513	Czech Republic	0.667	0.630
Czech Republic	0.167	−0.241	Romania	0.583	0.123
Poland	−0.111	−0.007	Hungary	0.333	−0.552

^1^ The means of values registered over the two periods.

## Data Availability

Not applicable.
